# Sex-Specific Differences in Patients with Hypertrophic Cardiomyopathy: A Cohort Study from Vienna

**DOI:** 10.3390/jpm16010056

**Published:** 2026-01-21

**Authors:** Christopher Mann, Rodi Tosun, Shehroz Masood, Theresa M. Dachs, Franz Duca, Christina Binder-Rodriguez, Christian Hengstenberg, Marianne Gwechenberger, Thomas A. Zelniker, Daniel Dalos

**Affiliations:** 1 Department of Medicine II, Division of Cardiology, Medical University of Vienna, 1090 Vienna, Austria; 2 Department of Bioimaging and Image-Guided Therapy, Division of Cardiovascular and Interventional Radiology, Medical University of Vienna, 1090 Vienna, Austria

**Keywords:** hypertrophic cardiomyopathy, sex differences, heart failure with preserved ejection fraction, diastolic dysfunction, obstructive hypertrophic cardiomyopathy

## Abstract

**Background**: Hypertrophic cardiomyopathy (HCM) is the most common inherited cardiovascular disease and affects male patients more often than women. Prior studies, however, suggested that women are diagnosed later and at advanced stages of the disease, present with more pronounced symptoms, and experience worse outcomes. **Objectives**: To investigate sex-specific differences in clinical, laboratory, and comprehensive imaging characteristics in a contemporary cohort of HCM patients from a tertiary referral center in Austria. **Methods**: We retrospectively analyzed 321 HCM patients enrolled in a prospective registry (2018–2024). All patients underwent a comprehensive baseline evaluation, including medical history, laboratory assessment, transthoracic echocardiography, and cardiac magnetic resonance imaging. **Results**: At diagnosis, women were significantly older (62 vs. 53 years, *p* < 0.001) and presented with more advanced functional class (NYHA ≥ II: 80% vs. 49%, *p* < 0.001). Six-minute walking distance was lower and obstructive HCM was more prevalent in women (425 vs. 505 m, *p* < 0.001, and 55% vs. 32%, *p* < 0.001, respectively). Echocardiographic assessment revealed higher diastolic filling pressures (E/E′ 18 vs. 10, *p* < 0.001), larger indexed atrial volumes (29.5 vs. 26.6 mL/m^2^, *p* < 0.001), a higher left ventricular ejection fraction (70% vs. 62%, *p* < 0.001), and a larger indexed interventricular septal thickness in women (10.2 vs. 9.3 mm/m^2^, *p* = 0.004). Moreover, serum levels of NT-proBNP were significantly higher in women (760 vs. 338 pg/L, *p* < 0.001). **Conclusions**: Female patients with HCM were diagnosed at an older age, presented with more advanced symptoms, had higher rates of obstructive physiology, and a phenotype characterized by diastolic dysfunction and elevated biomarkers, closely resembling heart failure with preserved ejection fraction. Recognizing these sex-specific disparities is crucial in improving diagnostic awareness and individualized therapeutic management.

## 1. Introduction

Hypertrophic cardiomyopathy (HCM) is the most common inherited cardiovascular disease, characterized by left ventricular hypertrophy without evident secondary causes [1,2]. The disease follows an autosomal dominant inheritance pattern, which implies an equal genotype distribution among males and females [3]. However, clinical presentation, diagnostic timing, and disease progression often differ substantially between sexes, significantly influencing patient management and prognosis. Despite previous research recognizing that women face later diagnoses, more severe symptoms, and worse outcomes, a granular understanding of contemporary phenotypic differences remains incomplete [4,5,6,7,8].

Emerging evidence further suggests that women with HCM more frequently present with a phenotype closely resembling heart failure (HF) with preserved ejection fraction (HFpEF). This HFpEF-like presentation is characterized by preserved left ventricular systolic function. Still, pronounced symptoms such as exertional dyspnea and fatigue result from significant diastolic dysfunction and elevated left atrial (LA) pressures [4,9,10,11]. Several hypotheses have been proposed to explain these observed sex-specific differences, including potential diagnostic bias, hormonal influences (especially estrogen), differential myocardial responses to pressure overload, and variability in clinical recognition and therapeutic practices [6,12,13,14,15].

Therefore, the aim of this observational analysis was to examine sex-related differences in clinical, laboratory, and echocardiographic characteristics among women and men in a well-defined HCM cohort evaluated at a tertiary referral center in Austria. Although sex-specific differences in HCM have been reported, it remains unclear how these differences present in contemporary practice when patients undergo a standardized, multimodality baseline evaluation. Large registries provide important epidemiologic and outcome data, but diagnostic pathways and the depth of phenotyping are often heterogeneous across participating sites. In contrast, our analysis leverages a harmonized baseline work-up performed at first presentation in our tertiary HCM program—including structured clinical assessment, biomarkers, and protocolized imaging—to characterize sex-specific differences at presentation.

## 2. Materials and Methods

### 2.1. Study Design

This observational study was conducted as part of the prospective HCM registry at the Department of Medicine II, Division of Cardiology at the Medical University of Vienna, Austria, a tertiary referral center for HCM patients. The study was approved by the ethics committee of the Medical University of Vienna (#1278/2018, approved on 22 June 2018) and was done in compliance with the Declaration of Helsinki. All participants provided written informed consent prior to inclusion. To enhance methodological transparency, we provide a completed critical appraisal using the Joanna Briggs Institute (JBI) checklist as Supplementary Table S1.

### 2.2. Patient Population

Between 25 June 2018 and 27 June 2024, a total of 321 HCM patients were included. Eligibility criteria included a confirmed HCM diagnosis and a complete baseline clinical and imaging dataset. All patients underwent a predefined baseline registry evaluation using standardized operating procedures, including structured clinical history, 12-lead electrocardiography (ECG)/Holter assessment, laboratory profiling (including N-terminal pro brain natriuretic peptide (NT-proBNP)), and protocolized transthoracic echocardiography (TTE), with guideline-based measurements. Where available, cardiac magnetic resonance imaging (CMR) followed a standardized institutional protocol with consistent post-processing. This harmonized workflow was applied irrespective of sex to support comparability across groups.

### 2.3. Transthoracic Echocardiography

TTE was performed following standard guidelines using GE Vivid E95 and Vivid E9 machines (GE Healthcare, Wauwatosa, WI, USA). Postprocessing analysis was performed using EchoPAC software V. 203 (GE Healthcare). All measurements were performed according to current guidelines [16].

### 2.4. Cardiac Magnetic Resonance Imaging

CMR was performed on a 1.5 Tesla scanner (MAGNETOM Avanto Fit, Siemens Healthcare GmbH, Erlangen, Germany) following a standardized protocol, which has been previously described [17]. LGE was assessed following the administration of 0.1 mmol/kg gadobutrol (Gadovist, Bayer Vital GmbH, Leverkusen, Germany), given that the estimated glomerular filtration rate was >30 mL/min/1.73 m^2^. The extent of myocardial fibrosis detected by LGE was measured as a percentage of the left ventricular (LV) mass using the full width at half-maximum method in a dedicated software (Medis Suite MR, V. 4.0.92.2 Medis Medical Imaging, Leiden, The Netherlands).

### 2.5. Statistical Analysis

Baseline characteristics were summarized as median (Q1–Q3) for continuous variables and as number (percentage) for categorical variables. Differences between groups were compared using the Mann–Whitney U test for continuous variables, and the Chi-square test or Fisher’s exact test for categorical variables, as appropriate. A two-sided *p*-value of <0.05 was considered statistically significant. All statistical analyses were performed using R (version 4.0.4; R Foundation for Statistical Computing, Vienna, Austria).

## 3. Results

The study cohort comprised 321 patients diagnosed with HCM, including 119 females (37%). The median age of the entire cohort was 55 years (43–64). Overall, 34% had obstructive HCM, defined as a left ventricular outflow tract (LVOT) gradient > 30 mmHg. Functional limitation, categorized as NYHA functional class ≥ 2, was observed in 60% of patients. Median body mass index was 28.4 kg/m^2^ (25.1–31.7), and median six-minute walk distance (6MWD) was 486 m (411–557). Common comorbidities across the total cohort included hypertension (56%), coronary artery disease (18%), type 2 diabetes mellitus (16%), and atrial fibrillation (23%). The median serum NT-proBNP level was 450 pg/L (153–1180). Serum levels of NT-proBNP were significantly higher in females (760 pg/L [370–1784] vs. 338 pg/L [101–927], *p* < 0.001). as well as serum levels of creatinine (13.3 g/dL [12.3–14.3] vs. 14.9 g/dL [13.7–15.85], *p* < 0.001 and 0.81 mg/dL [0.73–0.95] vs. 0.97 mg/dL [0.86–1.15], *p* < 0.001, respectively) (Table 1).

**Figure 1:**
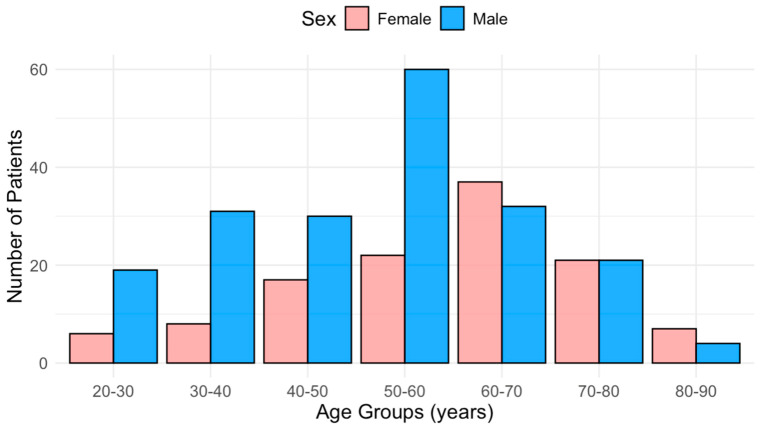
Age Distribution at referral by Sex.

**Figure 2:**
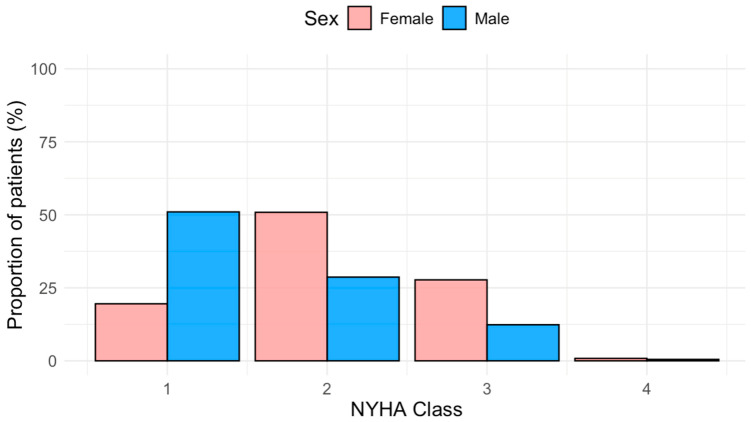
Relative NYHA Class Distribution by Sex.

Women primarily presented at a significantly older age than men (62 [48–69] vs. 53 [39–61] years, *p* < 0.001, Figure 1). Furthermore, functional status was significantly worse in women (NYHA ≥ II 80% vs. 49%, *p* < 0.001; Figure 2). Functional capacity, measured by the 6MWD, was significantly lower in female patients (425 m [392–490] vs. 505 m [436–588], *p* < 0.001). Obstructive HCM was more prevalent among females (55% vs. 32%; *p* < 0.001), as well as the presence of hypertension and the prescribed use of diuretic agents (65% vs. 50%, *p* = 0.034 and 28% vs. 11%, *p* = 0.032, respectively).

Regarding echocardiographic parameters, female patients showed a significantly impaired diastolic function, reflected by higher median E/E′ ratios (18 [14–22] vs. 10 [9–13], *p* < 0.001). In line with this, indexed LA volumes were significantly larger in females (29.5 cm^3^/m^2^ [27.0–32.1] vs. 26.6 cm^3^/m^2^ [23.5–29.6], *p* < 0.001) as well as indexed right atrial volumes (27.1 cm^3^/m^2^ [25.4–30.1] vs. 24.7 cm^3^/m^2^ [22.6–28.1], *p* < 0.001). Moreover, left ventricular ejection fraction (LVEF) among females was higher (70% [64–75] vs. 62% [55–67], *p* < 0.001) with lower LV end-diastolic dimensions (40 mm [35–43] vs. 44 mm [39–48], *p* < 0.001). While absolute interventricular septal (IVS) thickness was comparable between groups, the IVS indexed to body surface area (BSA) was significantly higher in females (10.17 mm/m^2^ [8.78–11.81] vs. 9.26 mm/m^2^ [8.16–10.89], *p* = 0.004). Extra cellular volume measured by CMR (29.9% [27.1–31.7] vs. 28.2% [26.0–29.7], *p* = 0.085) and the presence of LGE (49% vs. 55%, *p* = 0.50) were comparable between the groups (Table 2).

## 4. Discussion

In this analysis of a well-defined tertiary center HCM cohort in Austria, we identified distinct sex-specific differences in patient profiles, reinforcing and extending findings from previous studies. Female patients in our study represented only about one-third of the entire cohort and were primarily diagnosed with HCM approximately a decade later than their male counterparts. At this time point, women clinically presented with more advanced HF symptoms, more frequent obstructive HCM phenotypes, and more advanced diastolic dysfunction. This study addresses a practical knowledge gap: characterization of sex-specific differences at the first comprehensive tertiary-center evaluation using a standardized baseline workflow that integrates symptoms, functional capacity, biomarkers, and protocolized imaging. While prior studies have established that women are often diagnosed later and experience worse heart failure–related morbidity, fewer reports provide comparably harmonized phenotyping at the point of specialist consultation.

Our findings are in line with the emerging evidence on sex-specific differences in HCM. The observed delay in the primary diagnosis of women—approximately 10 years later in our cohort—is consistent with prior reports noting a 6–13 year age gap between female and male HCM patients [4,5,6,7,8]. Multiple studies have suggested that several biological and systemic factors contribute to this diagnostic delay. Biologically, premenopausal women may experience a slower progression of hypertrophy, potentially due to the modulatory effects of estrogen on myocardial remodeling [14,15]. Indeed, in our cohort, the peak prevalence of women occurred after the age of 60, corresponding to the postmenopausal period. Systemically, women have historically experienced under-recognition of cardiovascular diseases, including HCM, with less frequent referrals for further clinical investigation of murmurs or exercise intolerance, e.g., [4,5,6,7,8]. Our data support these notions, given the disproportionate number of men diagnosed in younger age groups. Already in 2005, Olivotto et al. highlighted the need for a heightened clinical suspicion of HCM in women to facilitate earlier diagnosis and intervention. In their large multicenter cohort including 969 patients, women were diagnosed later and experienced worse HF–related outcomes compared to men [12] Systematic reviews [8] and large observational cohorts consistently indicate that women with HCM are diagnosed later and present with more advanced symptoms, with excess risk largely attributable to heart failure–related events rather than arrhythmic endpoints in many datasets. Our results support this overall direction of evidence and extend it by providing detailed, standardized phenotyping at baseline in a contemporary Central European tertiary-care setting.

The increased symptom severity observed in female patients at first presentation corresponds to the results of previous observations. Significantly more women in our cohort presented with NYHA classes ≥ II compared to men, similar to findings reported by Olivotto et al. and other international registries [6,9,12]. Elevated levels of serum NT-proBNP further substantiate these observations, reflecting greater hemodynamic stress and advanced HF phenotypes in women. Our findings clearly indicated elevated serum NT-proBNP levels in a more symptomatic female subgroup, consistent with an advanced stage of the disease.

A notable finding of our analysis was the significantly higher percentage of obstructive HCM phenotypes among women. The higher prevalence of relevant LVOT obstruction may contribute to the increased symptom burden, including exertional dyspnea, angina, or syncope. In addition, smaller LV dimensions and imbalances of fluid status might predispose them to more dynamic outflow obstruction, which is supported by our findings of an increased intake of diuretic agents in female HCM patients. Whether hormonal factors, such as estrogen, have a decisive impact on myocardial and valvular tissues remains uncertain and merits dedicated studies [14,15]. Nevertheless, these findings underscore the importance of proactively identifying and managing obstructive physiology in female HCM patients at early stages, with implications for therapeutic choices including beta-blockers, myosin inhibitors, and/or invasive septal reduction therapies.

In accordance with more obstructive phenotypes, another interesting aspect was the evidence of a significantly impaired diastolic function among female patients, reflected by higher E/E′ ratios and larger indexed LA volumes. Literature supports that a combination of hypertrophy and age-related myocardial stiffening results in restrictive physiology in women, further contributing to a higher HF incidence [4,9]. Such observations align with the broader HFpEF phenotype commonly observed in older women [18]. Our female cohort effectively represents an “HCM-HFpEF overlap”, suggesting that clinical management strategies in HFpEF could be particularly relevant for this distinct patient collective with potential implications on long-term clinical outcomes.

While our study did not evaluate longitudinal outcomes due to its cross-sectional design, existing literature strongly supports the prognostic implications of our results. Advanced disease stage at baseline presentation in female HCM patients likely portends worse clinical outcome over time, aligning with reports of increased HF progression and mortality among women [10,11,13,19,20,21,22,23]. While previous studies did not consistently show sex-specific differences in the risk of sudden cardiac death [11], women experienced elevated cardiovascular mortality predominantly due to HF and stroke. Our cohort displayed similar implantable cardioverter-defibrillator implantation rates between women and men, which may reflect a comparable risk of malignant arrhythmias and further highlights that sex-specific disparities predominantly involve HF-related morbidity. Therefore, vigilant management of HF in female HCM patients and proactive early diagnosis may significantly influence long-term outcomes.

Our findings might offer several practical clinical implications. First, clinicians should maintain heightened suspicion for HCM in symptomatic women, particularly postmenopausal patients presenting with dyspnea or reduced exercise capacity, thereby minimizing the risk of undiagnosed entities. Second, following diagnosis, detailed assessment of HF indicators—particularly serum levels of NT-proBNP and diastolic functional parameters—may guide clinical risk stratification and therapeutic management. Lastly, our data may support the revision of diagnostic criteria tailored to sex-specific differences, advocating lower or indexed thresholds for hypertrophy in women in order to facilitate an earlier and more accurate diagnostic approach.

This study has several limitations, including its single-center, retrospective design, which may introduce selection bias and affect generalizability. While our findings highlight significant sex-specific differences and align with hypotheses concerning hormonal influences and diagnostic biases, our current dataset lacks the mechanistic information, including hormone levels, menopausal status, or detailed referral pathways, to definitively investigate these contributing factors. Although our cohort included a moderate number of female patients (n = 119), subgroup analyses may remain underpowered for specific comparisons. Whether the observed differences reflect delayed diagnosis/referral, sex-specific biological remodeling, or both cannot be definitively determined from this cross-sectional baseline analysis. We lacked granular data on symptom onset and referral pathways. Future work should integrate time-to-diagnosis metrics and apply age-matched and multivariable approaches to better disentangle diagnostic delay from sex-specific phenotype. Additionally, while phenotypic differences were the focus, potential genotypic influences warrant further exploration.

In conclusion, female HCM patients in this cohort presented at an older age, exhibited more advanced symptoms, and had a higher prevalence of obstructive physiology with significant diastolic dysfunction and HF characteristics resembling a HFpEF-phenotype. Recognizing these sex-specific disparities is essential in terms of an individualized diagnostic approach, enhanced clinical risk assessment, and early administration of, e.g., pharmacologic treatment aiming for improved clinical outcomes in affected patients.

## Figures and Tables

**Table 1:** Baseline Characteristics.

	Overall (n = 321)	Female (n = 119)	Male (n = 202)	*p*-Value
**Clinical parameters**				
Age, years	55 (43–64)	62 (48–69)	53 (39–61)	**<0.001**
Body mass index, kg/m^2^	28.4 (25.1–31.7)	28.0 (24.1–32.0)	28.4 (25.9–31.6)	0.40
Body surface area, m^2^	1.99 (1.85–2.15)	1.87 (1.69–1.95)	2.08 (1.96–2.22)	**<0.001**
NYHA functional class > = 2, n (%)	194 (60%)	95 (80%)	99 (49%)	**<0.001**
6-MWD, m	486 (411–557)	425 (392–490)	505 (436–588)	**<0.001**
ESC-SCD-risk score, %	2.87 (2.08–4.59)	2.75 (2.02–4.59)	2.92 (2.21–4.12)	0.8
Known pathogenic sarcomeric mutation, n (%)	14 (4.3%)	5 (4.2%)	9 (4.4%)	0.9
Prior ICD implantation, n (%)	25 (11%)	22 (11%)	3 (10%)	0.93
History of syncope, n (%)	56 (17%)	18 (15%)	34 (17%)	0.5
Coronary artery disease, n (%)	58 (18%)	17 (14%)	41 (20%)	0.20
Arterial hypertension, n (%)	179 (56%)	77 (65%)	102 (50%)	**0.034**
Diabetes mellitus type 2, n (%)	51 (16%)	21 (18%)	30 (15%)	0.81
Atrial fibrillation, n (%)	74 (23%)	29 (24%)	45 (22%)	0.8
Prior myectomy, n (%)	16 (5%)	7 (5.9%)	9 (4.5%)	0.6
Prior PTSMA, n (%)	26 (8.2%)	14 (12%)	12 (6.0%)	0.069
**Medication**				
Beta blocker, n (%)	202 (66%)	82 (65%)	120 (72%)	0.2
ACE inhibitor, n (%)	62 (20%)	18 (20%)	44 (17%)	0.2
Angiotensin receptor blocker, n (%)	93 (29%)	39 (30%)	54 (24%)	0.56
Alpha blocker, n (%)	30 (10%)	12 (11%)	18 (3.4%)	0.31
Diuretic agent, n (%)	34 (13%)	17 (11%)	17 (28%)	**0.032**
Oral anticoagulation, n (%)	57 (18%)	23 (18%)	34 (6.9%)	0.8
Antiplatelet agent, n (%)	75 (24%)	22 (18%)	53 (26%)	0.086
**Laboratory parameters**				
Hb, g/dL	14.30 (13.00–15.30)	13.30 (12.30–14.30)	14.90 (13.70–15.85)	**<0.001**
Creatinine, mg/dL	0.92 (0.79–1.09)	0.81 (0.73–0.95)	0.97 (0.86–1.15)	**<0.001**
eGFR, mL/min/1.73 m^2^	78 (58–96)	76 (53–96)	79 (58–96)	0.6
CRP, mg/dL	0.19 (0.09–0.40)	0.21 (0.11–0.42)	0.17 (0.08–0.39)	0.059
hsTnT, ng/L	14 (9–24)	13 (9–21)	14 (9–26)	0.6
NT-proBNP, pg/L	450 (153–1180)	760 (370–1784)	338 (101–927)	**<0.001**

Continuous data are reported as median (Q1–Q3). Bold texts in the *p*-Value column indicate statistical significance with *p* < 0.05. 6-MWD, 6 min walk test; Hb, Hemoglobin; CRP, C-reactive protein; eGFR, estimated glomerular filtration rate; ESC-SCD-risk score, European Society of Cardiology sudden cardiac death risk score; hsTnT, high-sensitivity Troponin T; NYHA, New York Heart Association; ICD, implantable cardioverter defibrillator; PTSMA, Percutaneous transluminal septal myocardial ablation; ACE: Angiotensin Converting Enzyme; NT-proBNP: N-terminal pro brain natriuretic peptide.

**Table 2:** Imaging Characteristics.

	Overall (n = 321)	Female (n = 119)	Male (n = 202)	*p*-Value
LA volume index, cm^3^/m^2^	27.5 (24.0–30.7)	29.5 (27.0–32.1)	26.6 (23.5–29.6)	**<0.001**
LA, mm	55 (50–61)	55 (50–59)	55 (50–63)	0.8
RA volume index, cm^3^/m^2^	25.8 (23.3–29.0)	27.1 (25.4–30.1)	24.7 (22.6–28.1)	**<0.001**
RA, mm	51 (47–56)	51 (47–55)	51 (48–57)	0.2
RV volume index, cm^3^/m^2^	15.34 (13.97–17.37)	16.08 (14.35–17.71)	15.24 (13.70–17.11)	**0.029**
RV ejection fraction, %	57 (51–65)	59 (53–67)	55 (49–62)	**0.013**
Interventricular septum, mm	19.0 (17.0–22.0)	19.0 (16.0–22.0)	19.0 (17.0–22.0)	0.2
Interventricular septum index, mm/m^2^	9.47 (8.29–11.17)	10.17 (8.78–11.81)	9.26 (8.16–10.89)	**0.004**
LV volume index, cm^3^/m^2^	21.1 (18.8–23.3)	21.3 (18.8–23.6)	21.1 (18.8–23.1)	0.4
LV, mm	42 (38–47)	40 (35–43)	44 (39–48)	**<0.001**
LVOT gradient >30 mmHg, n (%)	131 (41%)	65 (55%)	66 (32%)	**<0.001**
LVOT gradient, mmHg	25 (15–45)	44 (25−60)	20 (15–45)	**0.007**
LV ejection fraction, %	65 (58–70)	70 (64–75)	62 (55–67)	**<0.001**
Global longitudinal strain, %	−14.4 (−17.0–12.1)	−14.0 (−16.8–12.3)	−14.7 (−17.5–12.0)	0.6
E/A	0.97 (0.74–1.19)	0.93 (0.80–1.11)	0.99 (0.70–1.20)	0.8
E/E′	12 (10–17)	18 (14–22)	10 (9–13)	**<0.001**
LV stroke volume index, mL/m^2^	52 (46–62)	52 (45–62)	52 (47–62)	0.8
Cardiac output index, L/m^2^	3.33 (2.87–3.96)	3.37 (2.84–4.01)	3.30 (2.88–3.95)	>0.9
Extra cellular volume, %	28.5 (26.5–30.8)	29.9 (27.1–31.7)	28.2 (26.0–29.7)	0.085
Presence of LGE, n (%)	75 (53%)	23 (49%)	52 (55%)	0.5

Continuous data are reported as median (Q1–Q3). Bold texts in the *p*-Value column indicate statistical significance with *p* < 0.05. LA, left atrium; LGE, late gadolinium enhancement; LV, left ventricle; LVOT, left ventricular outflow tract; RA, right atrium; RV, right ventricle; E/A: Ratio of early (E) to late (A) diastolic transmitral flow velocities; E/E′: Ratio of early diastolic transmitral flow velocity (E) to early diastolic mitral annular tissue velocity (E′).

## Data Availability

The original contributions presented in this study are included in the article and Supplementary Materials. Further inquiries can be directed to the corresponding author.

## Supplementary Materials

The following supporting information can be downloaded at: https://www.mdpi.com/article/10.3390/jpm16010056/s1, Table S1: JBI Critical Appraisal Checklist–Analytical Cross-Sectional Studies.

## Abbreviations

The following abbreviations are used in this manuscript:

BSA	body surface area
CMR	cardiac magnetic resonance imaging
ECG	electrocardiography
HCM	hypertrophic cardiomyopathy
HF	heart failure
HFpEF	heart failure with preserved ejection fraction
IVS	interventricular septum
LA	left atrial
LV	left ventricular
LVEF	left ventricular ejection fraction
LVOT	left ventricular outflow tract
NT-proBNP	N-terminal pro brain natriuretic peptide
TTE	transthoracic echocardiography
