# Supplementary material for: Sex-Specific Differences in Patients with Hypertrophic Cardiomyopathy: A Cohort Study from Vienna
Source: J Pers Med. 2026 Jan 21;16(1):56. doi: 10.3390/jpm16010056 (PMC12842707; doi:10.3390/jpm16010056)
Supplement: Supplementary file 1 [file jpm-16-00056-s001.zip › jpm-4064017-supplementary.pdf]

Supplementary Table S1. JBI Critical Appraisal Checklist – Analytical Cross-Sectional Studies

JBI item	Judgment (Yes/No/Unclear/NA)	Justification for this study
1. Were the criteria for inclusion in the sample clearly defined?	**Yes**	Inclusion/exclusion criteria are stated (confirmed HCM diagnosis; adult patients; registry enrolment within 2018–2024; complete baseline dataset required for analysis).
2. Were the study subjects and the setting described in detail?	**Yes**	Setting is a tertiary referral HCM program at the Medical University of Vienna; recruitment period and cohort size (n=321; 37% women) are reported; baseline assessments described.
3. Was the exposure measured in a valid and reliable way? *(Exposure: sex)*	**Yes**	Sex was recorded as a routine clinical/registry variable at baseline using standardized intake documentation, applied consistently across participants.
4. Were objective, standard criteria used for measurement of the condition? *(Condition: HCM)*	**Yes**	HCM diagnosis was established clinically and confirmed at a tertiary center using standard diagnostic definitions; baseline imaging followed guideline-based measurement principles as stated in Methods.
5. Were confounding factors identified?	**Yes**	Key potential confounders are acknowledged (e.g., age at presentation, body size/BSA, comorbidities such as hypertension) and are explicitly discussed as factors that may contribute to sex differences.
6. Were strategies to deal with confounding factors stated?	**No**	The primary analyses are unadjusted (group comparisons). The manuscript discusses confounding as a limitation and outlines future approaches (e.g., age-matched or multivariable analyses), but no formal adjustment strategy is implemented in the current analysis.
7. Were the outcomes measured in a valid and reliable way?	**Yes**	Outcomes/variables (NYHA class, 6MWD, NT-proBNP, LVOT gradient, LV dimensions, IVS index) were collected within a standardized in-center baseline workflow and measured using routine clinical methods and protocolized imaging acquisition/post-processing as described.
8. Was appropriate statistical analysis used?	**Yes**	Data are summarized as median (Q1–Q3) and n (%); between-group comparisons use Mann–Whitney U for continuous variables and χ^2/Fisher's exact tests for categorical variables, consistent with distributional assumptions and data type.

JBI, Joanna Briggs Institute. This manuscript reports a cross-sectional baseline analysis (retrospective analysis of prospectively collected registry data)
